# Supplementary material for: TRPM7 kinase-mediated immunomodulation in macrophage plays a central role in magnesium ion-induced bone regeneration
Source: Nat Commun. 2021 May 17;12:2885. doi: 10.1038/s41467-021-23005-2 (PMC8128914; doi:10.1038/s41467-021-23005-2)
Supplement: Supplementary file 1 — Supplementary Information [file 41467_2021_23005_MOESM1_ESM.pdf]

Supplementary Information

TRPM7 kinase-mediated immunomodulation in macrophage plays a central role in magnesium ion-induced bone regeneration

Wei Qiao [1,2,3], Karen H.M. Wong [1,2], Jie Shen [1,2], Wenhao Wang [1,2], Jun Wu [1,2], Jinhua Li [1,2,4], Zhengjie Lin [1,2], Zetao Chen [5,6], Jukka P. Matinlinna [3], Yufeng Zheng [7], Shuilin Wu [8], Xuanyong Liu [9], Keng Po Lai [10, 11], Zhuofan Chen [6*], Yun Wah Lam [11*], Kenneth M.C. Cheung [1,2], Kelvin W.K. Yeung [1,2,12*]

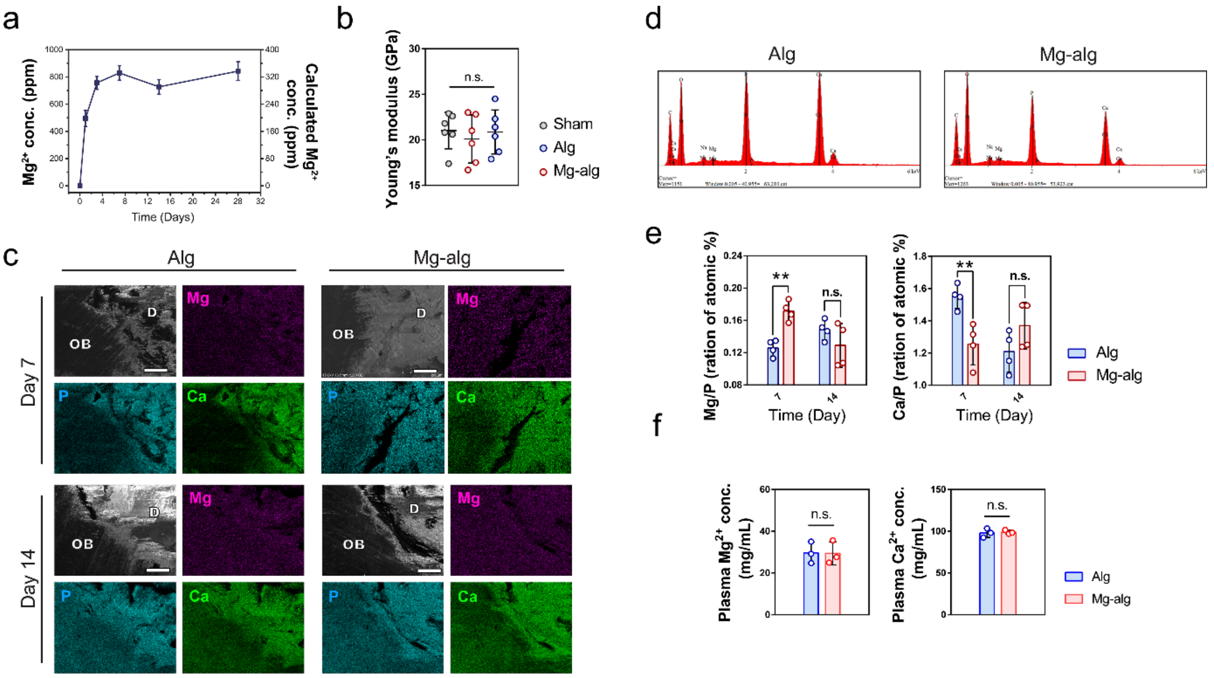

Supplementary Fig. 1: (a) Cumulative Mg^{2+} released from the Mg crosslinked alginate measured *in vitro* using ICP-OES. **(b)** Young's modulus of newly formed bone in the sham group (n = 6), alginate group (n = 6) and Mg-alginate group (n = 6). **(c)** Representative SEM images and EDX mapping of Mg, P and Ca in and around the rat femoral defects grafted with alginate or Mg-releasing alginate 7 and 14 days after the operation (n=3), scale bars = 200 μm. (OB: old bone, D: defect) **(d)** Representative SEM-EDX spectrums showing the atomic composition in the femur defects grafted with alginate or Mg-releasing alginate 7 days after the operation. **(e)** Quantitative data showing the Mg/P and CaP ratio in defect areas grafted with alginate or Mg-releasing alginate on day 7 and day 14 (n=4). **(f)** The serum concentration of Mg^{2+} and Ca^{2+} measured using ICP-OES (n=3). Data are mean ± s.d. n.s. P>0.05, *P<0.05, **P<0.01 by one-way ANOVA with Tukey's *post hoc* test.

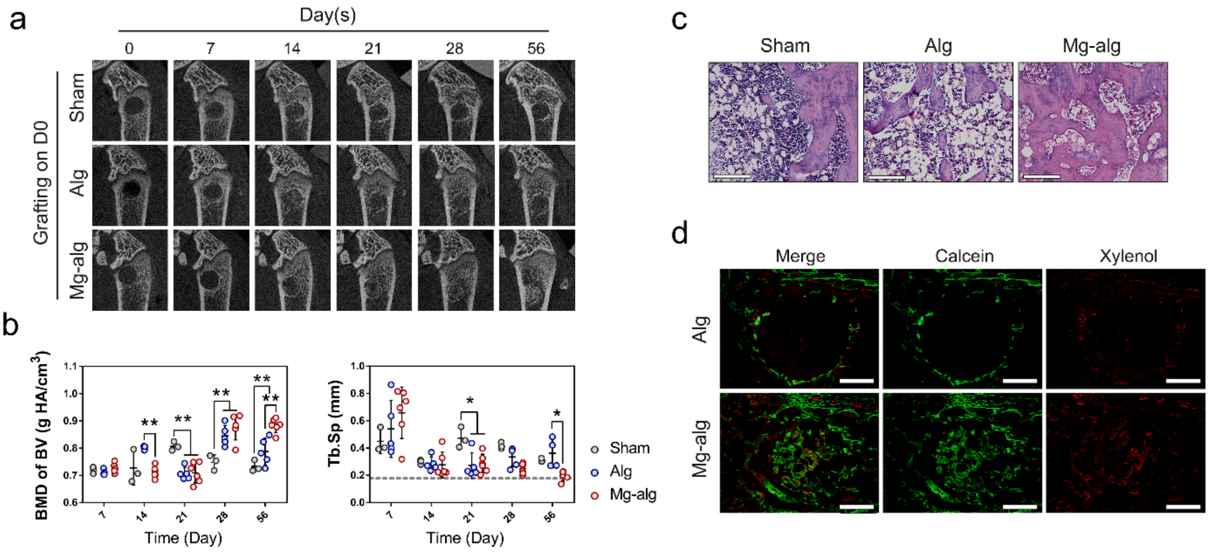

Supplementary Fig. 2: (a, b) Representative micro-CT images **(a)** and corresponding measurements of BMD of BV and Tb.Sp. **(b)** showing the healing process of rat femoral defects without grafting (Sham group), grafted with pure alginate (serve as a control) or Mg-releasing alginate. Sham, n=3; Alg, n=5; Mg-alg, n=6. Data are mean ± s.d. n.s. P>0.05, *P<0.05, **P<0.01 by one-way ANOVA with Tukey's *post hoc* test. **(c)** Representative H&E staining of the defects in rat femur on day 56 (n=3), scale bars =200 µm. **(d)** Representative images of calcein/xylenol labeling for bone regeneration in the rat femoral defects grafted with alginate or Mg crosslinked alginate (n=6), scale bars = 1 mm.

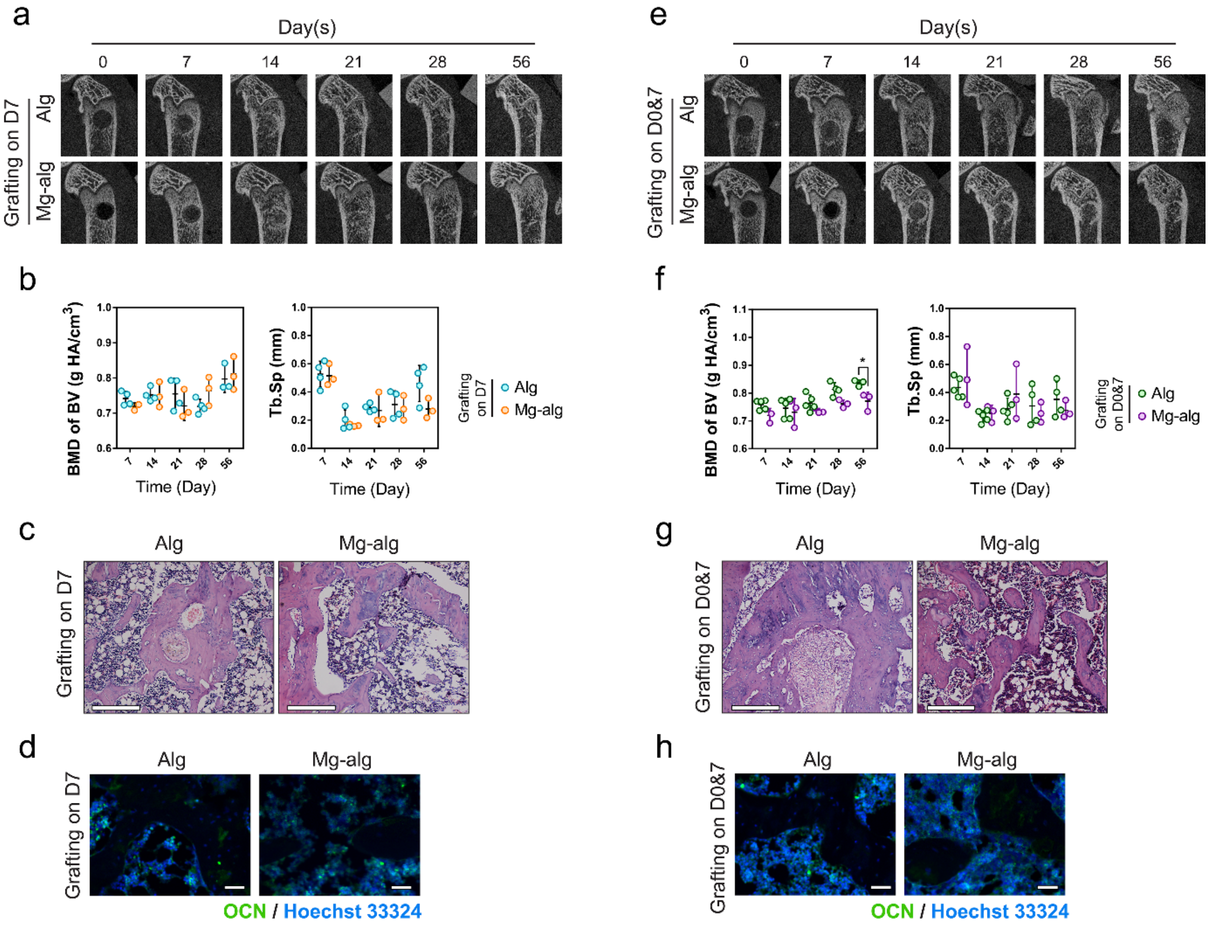

Supplementary Fig. 3: (a) Representative micro-CT images **(a)** and Corresponding measurements of BMD of BV and Tb.Sp **(b)** showing the healing process of rat femoral defects when pure alginate (serve as a control) or Mg crosslinked alginate were grafted on Day 7 after the operation. Alg, n=4; Mg-alg, n=3. Data are mean ± s.d. n.s. $P>0.05$, *$P<0.05$, **$P<0.01$ by one-way ANOVA with Tukey's *post hoc* test. **(c, d)** Representative H&E staining **(c,** n=3, scale bars =200 µm**)** and immunofluorescent images showing the expression of OCN **(d,** n=3, scale bars =100 µm**)** in rat femur defect when the material was grafted on Day 7 after the operation. **(e, f)** Representative micro-CT images **(e)** and corresponding measurements of BMD of BV and Tb.Sp. **(f)** showing the healing process of rat femoral defects when pure alginate (serve as a control) or Mg crosslinked alginate were grafted both immediately and 7 days after the operation. Alg, n=4; Mg-alg, n=3. **(g, h)** Representative H&E staining **(g,** n=3, scale bars =200 µm**)** and immunofluorescent images showing the expression of OCN **(h,** n=3, scale bars =100 µm**)** in rat femur defect when the material was grafted immediately and 7 days after the operation.

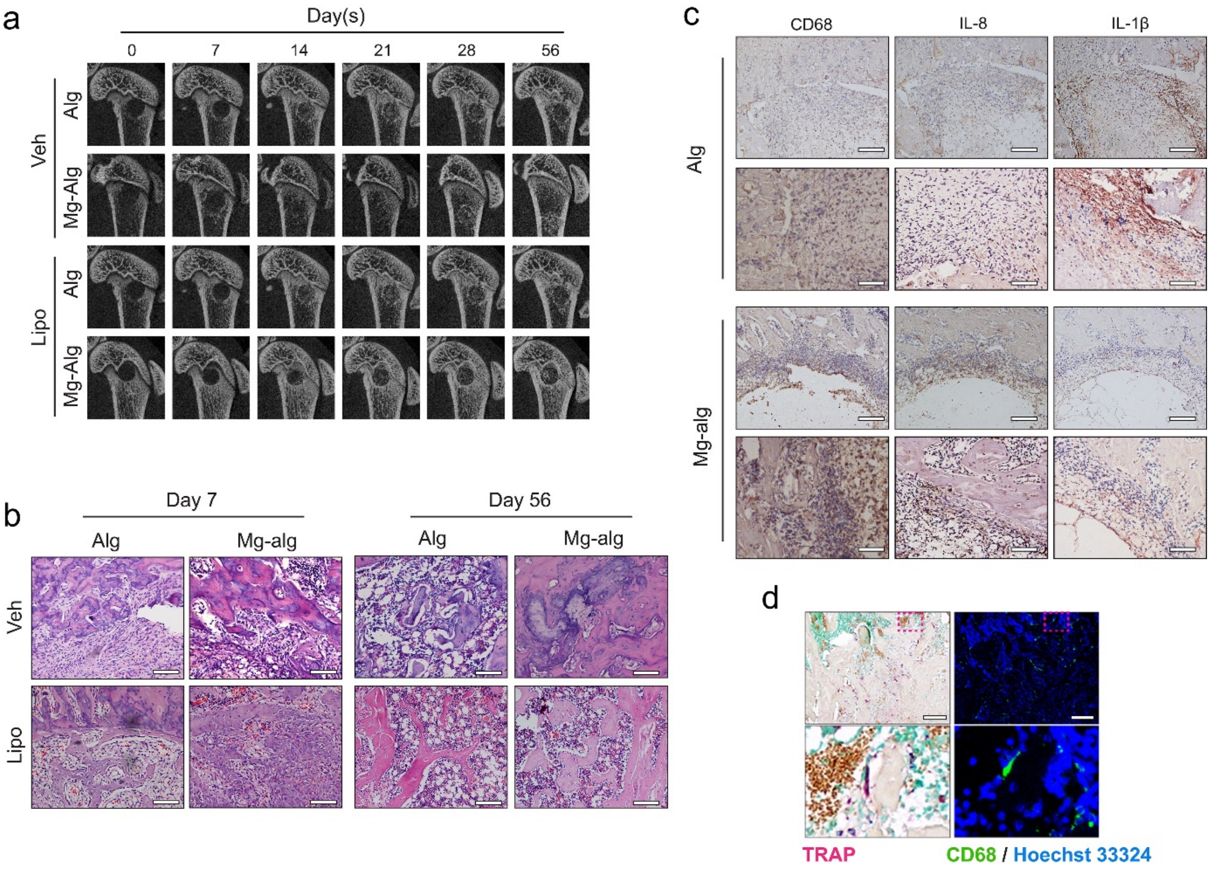

Supplementary Fig. 4: (a) Representative micro-CT images showing the healing process of femoral defects grafted with pure alginate (n = 3) or Mg-releasing alginate (n = 3) in macrophage depleted rats compared with control group injected with vehicle from day 7 to day 56. **(b)** Representative H&E staining of the defects in rat femur on day 7 and day 56 in control rats and macrophage depleted rats (n=3), scale bars =200 μm. **(c)** Representative IHC images showing the expression of CD68, IL-8 and IL-1β within the rat femoral defects grafted with alginate or Mg-alginate on day 7 (n=3). Lower images (scale bar = 100 μm) are high-resolution versions of the upper images (scale bar = 200 μm). **(d)** Representative TRAP staining images (left) and corresponding immunofluorescent images (right) showing the expression of CD68 in $TRAP^+$ cells at day 7 after the operation (n=3). lower images are high-resolution versions of the boxed regions in the upper images (scale bars = 200 μm).

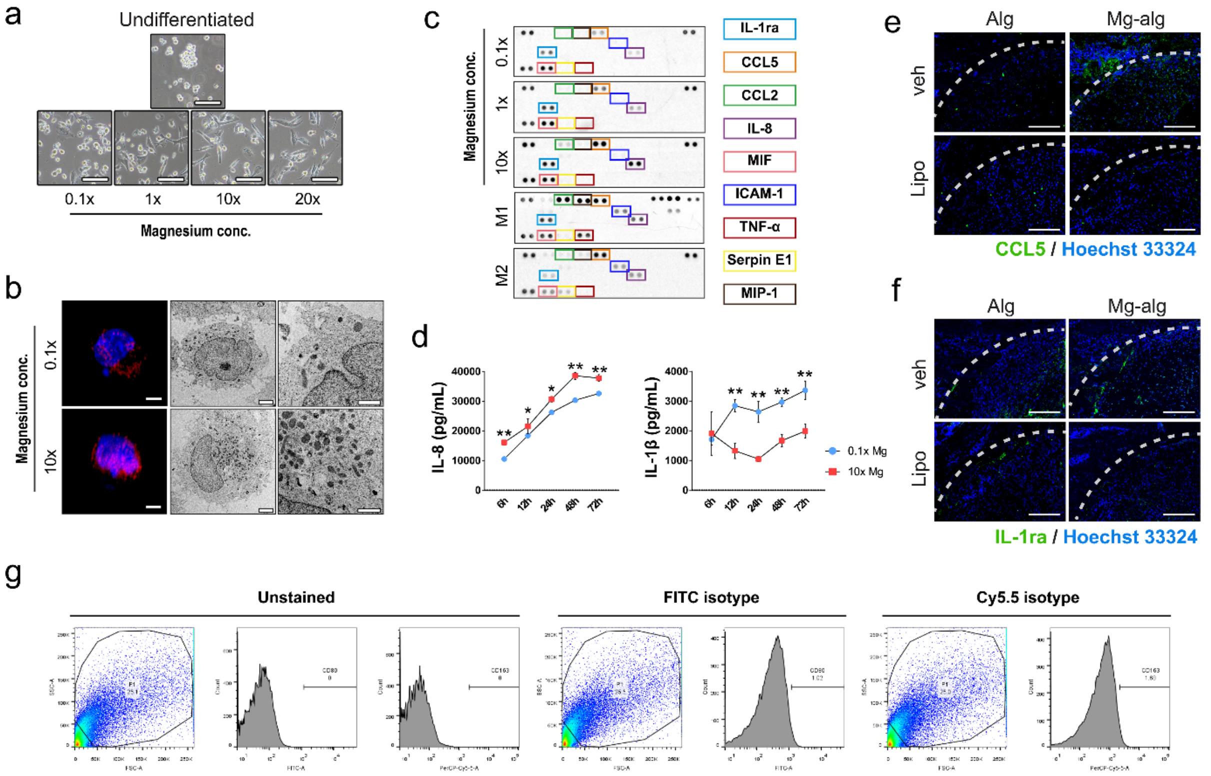

Supplementary Fig. 5: (a) Representative microscopy images showing changes in the morphology of THP-1 derived macrophages after PMA-induced differentiation with the addition of different concentrations of Mg^{2+} (n ≥ 5). **(b)** Representative 3D confocal (scale bar = 2 μm) and TEM images showing the increased number of mitochondria in THP1-derived macrophages after the stimulation of Mg^{2+} (n≥5). right images (scale bar = 500 nm) are high-resolution versions of the middle images (scale bar = 2 μm). **(c)** Cytokine array showing the major cytokines produced by Mg^{2+} treated macrophages, as well as classical differentiated M1 and M2 macrophages. **(d)** ELISA analysis showing the time-dependent effect of Mg^{2+} on the production of IL-8 and IL-1β in THP1-derived macrophages (n=3). Data are mean ± s.d. **P*<0.05, ***P*<0.01 by one-way ANOVA with Tukey's *post hoc* test. **(e, f)** Representative immunofluorescent images showing the expression of CCL5 **(a)** and IL-1ra **(b)** on day 7 in the grafted defects in the rat femora (n=3), scale bars =200 μm. **(g)** Gating strategies for flow cytometry data: the total THP1 cells were first gated by forward and side scatter, CD163, CD206 and CD80 positive cells were further gated using the same gating strategies defined by THP1 cells fixed identically to the experimental groups and stained with immunoglobulin-matched FITC- or Cy5.5-conjugated isotype control.

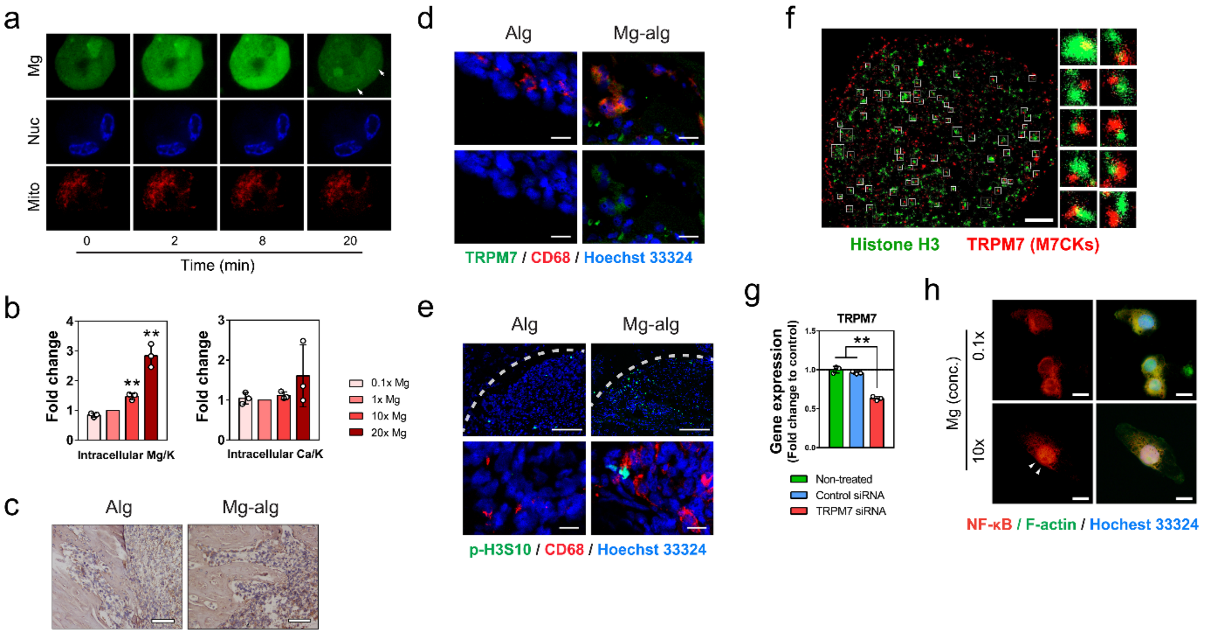

Supplementary Fig. 6: (a) Representative fluorescence images showing the entry of Mg^{2+} into macrophages, the accumulation of Mg^{2+} in nuclei, as well as the dynamic changes in mitochondria after the addition of 8 mM $MgCl_2$ (n=5). **(b)** ICP-OES measurement of intracellular Mg to K ratio and Ca to K ration in macrophages after the stimulation of different concentrations of Mg^{2+} (n=3). Data are mean ± s.d. n.s. $**P<0.01$ by one-way ANOVA with Tukey's *post hoc* test. **(c, d)** Representative IHC images **(c**, n=3, scale bars = 100 μm**)** and immunofluorescent images **(d**, n=3, scale bars = 20 μm**)** showing the upregulation of TRPM7 in macrophages in and around the defects grafted with Mg-releasing alginate 7 days after the surgery. **(e)** Representative immunofluorescent images showing phosphorylation of Histone H3S10 in macrophages in and around the defects grafted with Mg-releasing alginate 7 days after the surgery (n=3). lower images (scale bars = 20 μm**)** are high-resolution versions of the boxed regions in the upper images (scale bars = 500 μm). **(f)** Representative super-resolution images showing the colocalization of TRPM7 (M7CKs) and Histone H3 within the nucleus of macrophages. Scale bar = 1 μm. **(g)** The effects of TRPM7 siRNA on the expression of TRPM7 in THP1-derived macrophages (n=3). **(h)** Representative fluorescence images showing the nuclear translocation of NF-κB in macrophages after the stimulation of Mg^{2+} (n=3). Scale bars = 5 μm.

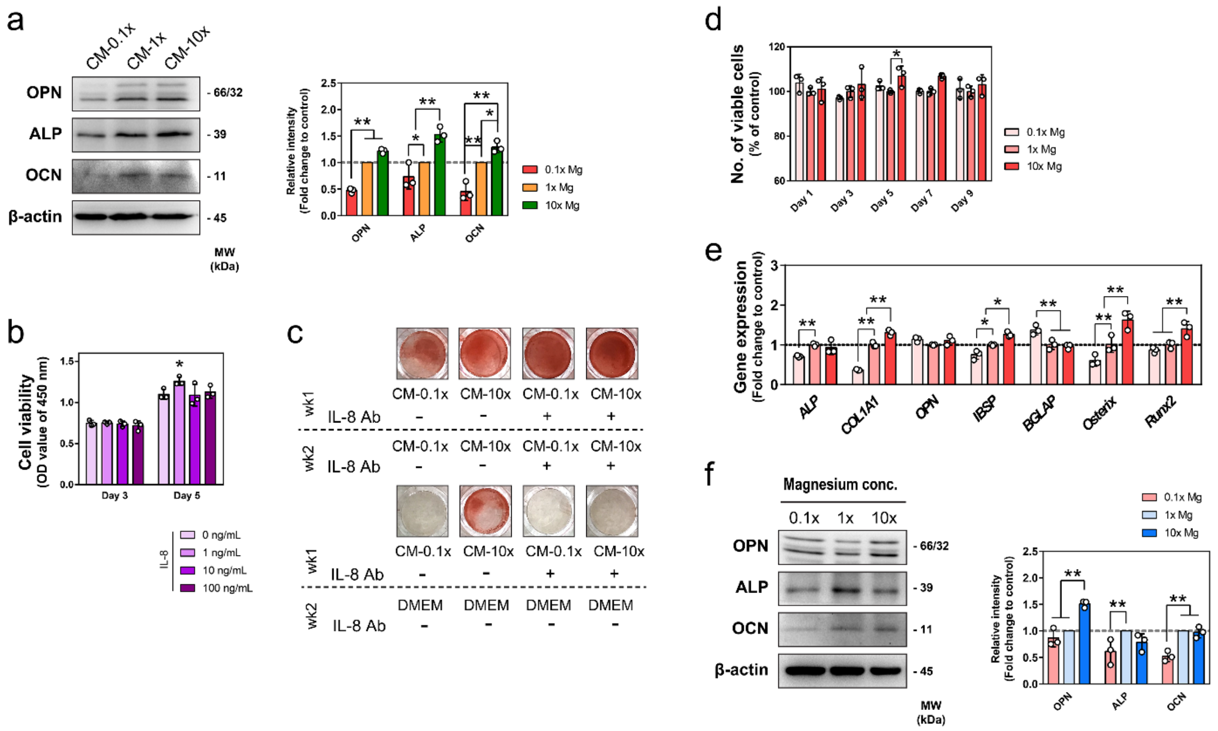

Supplementary Fig. 7: (a) Representative western blots and corresponding quantification showing the bone markers expression of MSC cultured in conditional medium from macrophages stimulated with different concentrations of Mg^{2+} (n=3). **(b)** The cell viability of MSC cultured in DMEM supplemented with different concentrations of recombinant IL-8 for 3 and 5 days (n=3). **(c)** Alizarin Red staining of mineralized nodules of MSC cultured in conditional medium from macrophages with or without the addition of IL-8 neutralizing antibody. **(d, e)** The number of viable cells (**d**, n=3**)** and the osteogenic related gene expression of MSC **(e**, n=3**)** cultured in DMEM supplemented with different concentrations of Mg^{2+}. **(f)** Representative western blots and corresponding quantification showing the bone markers expression of MSC cultured in DMEM supplemented with different concentrations of Mg^{2+} (n=3). Data are mean ± s.d. n.s. *P*>0.05, **P*<0.05, ***P*<0.01 by one-way ANOVA with Tukey's *post hoc* test.

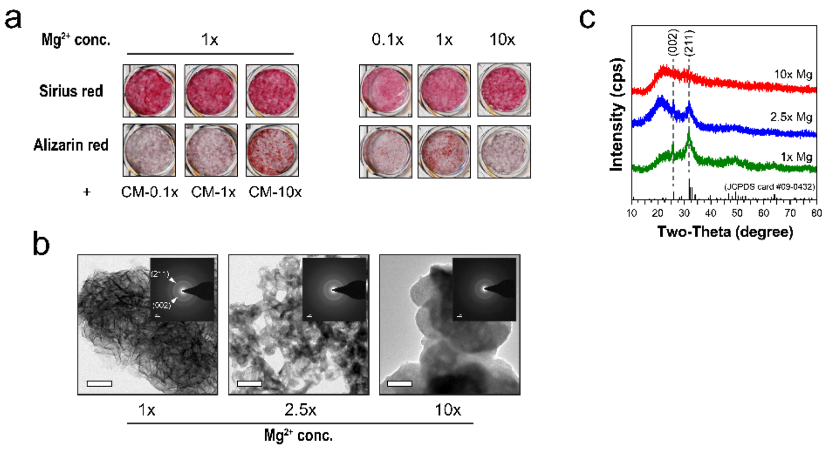

Supplementary Fig. 8: (a) Sirius red and alizarin red staining showing the ECM formation and mineralization of MSC treated with conditional medium from Mg^{2+}-stimulated macrophage or DMEM supplemented with different concentrations of Mg^{2+} for 7 days. **(b)** Representative TEM images and corresponding SAED patterns showing the precipitation formed in culture medium supplemented with different concentrations of Mg^{2+} scale bars = 50 nm (n=3). **(c)** XRD patterns of the precipitation formed in culture medium supplemented with different concentrations of Mg^{2+}, stoichiometric HAp pattern (JCPDS card #09-0432) was shown as a reference.

Supplementary Table 1. Primers used in the RT-qPCR assays.

Gene		Primer
hBMP2	Forward:	5'-ACCCGCTGTCTTCTAGCGT-3'
	Reverse:	5'-TTTCAGGCCGAACATGCTGAG-3'
hVEGFA	Forward:	5'-AGGGCAGAATCATCACGAAGT-3'
	Reverse:	5'-AGGGTCTCGATTGGATGGCA-3'
hM-CSF	Forward:	5'-TGGCGAGCAGGAGTATCAC-3'
	Reverse:	5'-AGGTCTCCATCTGACTGTCAAT-3'
hTNF-α	Forward:	5'-CCTCTCTCTAATCAGCCCTCTG-3'
	Reverse:	5'-GAGGACCTGGGAGTAGATGAG-3'
hIFN-γ	Forward:	5'-TCGGTAACTGACTTGAATGTCCA-3'
	Reverse:	5'-TCGCTTCCCTGTTTTAGCTGC-3'
hIL-1β	Forward:	5’-ATGATGGCTTATTACAGTGGCAA-3’
	Reverse:	5’-GTCGGAGATTCGTAGCTGGA-3’
hIL-10	Forward:	5’-TCAAGGCGCATGTGAACTCC-3’
	Reverse:	5’-GATGTCAAACTCACTCATGGCT-3’
hIL-1ra	Forward:	5’-TTCCTGTTCCATTCAGAGACGAT-3’
	Reverse:	5’-AATTGACATTTGGTCCTTGCAA-3’
hTGF-β1	Forward:	5’-GGCCAGATCCTGTCCAAGC-3’
	Reverse:	5’-GTGGGTTTCCACCATTAGCAC-3’
hTRPM7	Forward:	5’-ACTGGAGGAGTAAACACAGGT-3’
	Reverse:	5’-TGGAGCTATTCCGATAGTGCAA-3’
hOPG	Forward:	5’-CAACATATCGTTGGATCACAGCA-3’
	Reverse:	5’-GACAGACTCACTTTATGGGAACC-3’
hOSM	Forward:	5’-CACAGACTGGCCGACTTAGAG-3’
	Reverse:	5’-AGTCCTCGATGTTCAGCCCA-3’
hIL-8	Forward:	5’-TTTTGCCAAGGAGTGCTAAAGA-3’
	Reverse:	5’-AACCCTCTGCACCCAGTTTTC-3’
hCCL5	Forward:	5’-CCAGCAGTCGTCTTTGTCAC-3’
	Reverse:	5’-CTCTGGGTTGGCACACACTT-3’
hCD163	Forward:	5'-TTTGTCAACTTGAGTCCCTTCAC-3'
	Reverse:	5'-TCCCGCTACACTTGTTTTCAC-3'
hCD206	Forward:	5'-TCCGGGTGCTGTTCTCCTA-3'
	Reverse:	5'-CCAGTCTGTTTTTGATGGCACT-3'
hOsterix	Forward:	5'-CCTCTGCGGGACTCAACAAC-3'
	Reverse:	5'-AGCCCATTAGTGCTTGTAAAGG-3'
hRunx2	Forward:	5'-TGGTTACTGTCATGGCGGGTA-3'
	Reverse:	5'-TCTCAGATCGTTGAACCTTGCTA-3'
hCOL1A1	Forward:	5'-GAGGGCCAAGACGAAGACATC-3'
	Reverse:	5'-CAGATCACGTCATCGCACAAC-3'
hBGLAP	Forward:	5'-CACTCCTCGCCCTATTGGC-3'
	Reverse:	5'-CCCTCCTGCTTGGACACAAAG-3'
hSPP1	Forward:	5'-CTCCATTGACTCGAACGACTC-3'
	Reverse:	5'-CAGGTCTGCGAAACTTCTTAGAT-3'
hIBSP	Forward:	5'-CACTGGAGCCAATGCAGAAGA-3'
	Reverse:	5'-TGGTGGGGTTGTAGGTTCAAA-3'
hALP	Forward:	5'-AACATCAGGGACATTGACGTG-3'
	Reverse:	5'-GTATCTCGGTTTGAAGCTCTTCC-3'
hTRAP	Forward:	5'-GACTGTGCAGATCCTGGGTG-3'
	Reverse:	5'-GGTCAGAGAATACGTCCTCAAAG-3'
hRANK	Forward:	5'-AGATCGCTCCTCCATGTACCA-3'
	Reverse:	5'-GCCTTGCCTGTATCACAAACTTT-3'
hCTSK	Forward:	5'-ACACCCACTGGGAGCTATG-3'
	Reverse:	5'-GACAGGGGTACTTTGAGTCCA-3'
hCTR	Forward:	5'-CCTATCCAACAATAGAGCCCAAG-3'
	Reverse:	5'-TGCATTCGGTCATAGCATTTGTA-3'
hGAPDH	Forward:	5'-GGAGCGAGATCCCTCCAAAAT-3'
	Reverse:	5'-GGCTGTTGTCATACTTCTCATGG-3'
mBMP2	Forward:	5'-TCTTCCGGGAACAGATACAGG-3'
	Reverse:	5'-TGGTGTCCAATAGTCTGGTCA-3'
mVEGFA	Forward:	5'-GCACATAGAGAGAATGAGCTTCC-3'
	Reverse:	5'-CTCCGCTCTGAACAAGGCT-3'

mTNF-α	Forward：	5'-CAGGCGGTGCCTATGTCTC-3'
	Reverse：	5'-CGATCACCCCGAAGTTCAGTAG-3'
mIL-1β	Forward：	5’-GAAATGCCACCTTTTGACAGTG-3’
	Reverse：	5’-TGGATGCTCTCATCAGGACAG-3’
mIL-10	Forward：	5’-CTTACTGACTGGCATGAGGATCA-3’
	Reverse：	5’-GCAGCTCTAGGAGCATGTGG-3’
mIL-1ra	Forward：	5’-GCTCATTGCTGGGTACTTACAA-3’
	Reverse：	5’-CCAGACTTGGCACAAGACAGG-3’
mTGF-β1	Forward：	5’-CCACCTGCAAGACCATCGAC-3’
	Reverse：	5’-CTGGCGAGCCTTAGTTTGGAC-3’
mTRPM7	Forward：	5’-AGGATGTCAGATTTGTCAGCAAC-3’
	Reverse：	5’-CCTGGTTAAAGTGTTCACCCAA-3’
mOSM	Forward：	5’-CCCGGCACAATATCCTCGG-3’
	Reverse：	5’-TCTGGTGTTGTAGTGGACCGT-3’
mMIP-2	Forward：	5’-CCAACCACCAGGCTACAGG-3’
	Reverse：	5’-GCGTCACACTCAAGCTCTG-3’
mCCL5	Forward：	5’-TTTGCCTACCTCTCCCTCG-3’
	Reverse：	5’-CGACTGCAAGATTGGAGCACT-3’
mGAPDH	Forward：	5'- AGGTCGGTGTGAACGGATTTG-3'
	Reverse：	5'- TGTAGACCATGTAGTTGAGGTCA-3'
