# Supplementary material for: TRPM7 kinase-mediated immunomodulation in macrophage plays a central role in magnesium ion-induced bone regeneration
Source: Nat Commun. 2021 May 17;12:2885. doi: 10.1038/s41467-021-23005-2 (PMC8128914; doi:10.1038/s41467-021-23005-2)
Supplement: Supplementary file 3 — Reporting Summary [file 41467_2021_23005_MOESM3_ESM.pdf]

nature research

Corresponding author(s): NCOMMS-20-15243B

Last updated by author(s): Jan 27, 2021

Reporting Summary

Nature Research wishes to improve the reproducibility of the work that we publish. This form provides structure for consistency and transparency in reporting. For further information on Nature Research policies, see our Editorial Policies and the Editorial Policy Checklist.

Statistics

For all statistical analyses, confirm that the following items are present in the figure legend, table legend, main text, or Methods section.

n/a	Confirmed	
☐	☒	The exact sample size (*n*) for each experimental group/condition, given as a discrete number and unit of measurement
☐	☒	A statement on whether measurements were taken from distinct samples or whether the same sample was measured repeatedly
☐	☒	The statistical test(s) used AND whether they are one- or two-sided<br>*Only common tests should be described solely by name; describe more complex techniques in the Methods section.*
☐	☒	A description of all covariates tested
☐	☒	A description of any assumptions or corrections, such as tests of normality and adjustment for multiple comparisons
☐	☒	A full description of the statistical parameters including central tendency (e.g. means) or other basic estimates (e.g. regression coefficient) AND variation (e.g. standard deviation) or associated estimates of uncertainty (e.g. confidence intervals)
☐	☒	For null hypothesis testing, the test statistic (e.g. *F*, *t*, *r*) with confidence intervals, effect sizes, degrees of freedom and *P* value noted<br>*Give P values as exact values whenever suitable.*
☒	☐	For Bayesian analysis, information on the choice of priors and Markov chain Monte Carlo settings
☒	☐	For hierarchical and complex designs, identification of the appropriate level for tests and full reporting of outcomes
☒	☐	Estimates of effect sizes (e.g. Cohen's *d*, Pearson's *r*), indicating how they were calculated

Our web collection on statistics for biologists contains articles on many of the points above.

Software and code

Policy information about availability of computer code

Data collection	GraphPad Prism 7, Microsoft Excel, Adobe Photoshop CC, and Adobe Illustrator CC were used for performing general data analysis and generating figures. ImageJ and were used for quantitative imaging analyses.
Data analysis	GraphPad Prism 7, Microsoft Excel, Adobe Photoshop CC, and Adobe Illustrator CC were used for performing general data analysis and generating figures. ImageJ and were used for quantitative imaging analyses.

For manuscripts utilizing custom algorithms or software that are central to the research but not yet described in published literature, software must be made available to editors and reviewers. We strongly encourage code deposition in a community repository (e.g. GitHub). See the Nature Research guidelines for submitting code & software for further information.

Data

Policy information about availability of data

All manuscripts must include a data availability statement. This statement should provide the following information, where applicable:

- Accession codes, unique identifiers, or web links for publicly available datasets
- A list of figures that have associated raw data
- A description of any restrictions on data availability

All relevant data that support the findings of this study are available from the corresponding author upon reasonable requests.

Field-specific reporting

Please select the one below that is the best fit for your research. If you are not sure, read the appropriate sections before making your selection.

- [x] Life sciences
- [ ] Behavioural & social sciences
- [ ] Ecological, evolutionary & environmental sciences

For a reference copy of the document with all sections, see nature.com/documents/nr-reporting-summary-flat.pdf

Life sciences study design

All studies must disclose on these points even when the disclosure is negative.

Sample size	Each finding was confirmed with minimum necessary number such as 1-6 replicates for each experiments.
Data exclusions	N/A
Replication	All experiments were performed with independent replicates as described in the figure legends
Randomization	Randomization was performed blindly among animals
Blinding	N/A

Reporting for specific materials, systems and methods

We require information from authors about some types of materials, experimental systems and methods used in many studies. Here, indicate whether each material, system or method listed is relevant to your study. If you are not sure if a list item applies to your research, read the appropriate section before selecting a response.

Materials & experimental systems

n/a	Involved in the study	
	x	Antibodies
	x	Eukaryotic cell lines
x		Palaeontology and archaeology
	x	Animals and other organisms
x		Human research participants
x		Clinical data
x		Dual use research of concern

Methods

n/a	Involved in the study	
x		ChIP-seq
	x	Flow cytometry
x		MRI-based neuroimaging

Antibodies

Antibodies used

anti-OCN (ab93876, Abcam), anti-IL-8 (ab18672, ab7747, Abcam), rabbit anti-CCL5 (ab9783, Abcam), anti-IL-1β (ab9722, Abcam), anti-IL-1ra (ab124962, Abcam), anti-CD68 (ab125212, ab31630, Abcam), rabbit anti-Phospho-Histone H3S10 (ab5176, Abcam), anti-Histone H3 (ab1791, Abcam), anti-TRPM7 (ab135817, ab109438, ab85016, Abcam), rabbit anti-OPN (ab8448, Abcam), mouse anti-ALP (sc-365765, Santa Cruz), anti-IKKβ (#8943, CST, USA), anti-IKKα (#11930, CST), anti- NF-κB p65 (#8241, CST), anti-Phospho-IκBα (#2859, CST), anti-IκBα (#4814, CST), anti-Phospho-JNK (#4668, CST), rabbit anti-JNK (#9252, CST), anti-NFATc1 (sc-7294, Santa Cruz), and mouse anti-β-actin (#8457, CST)

Validation

https://www.abcam.com/osteocalcin-antibody-ab93876.html
https://www.abcam.com/il-8-antibody-807-ab18672.html
https://www.abcam.com/il-8-antibody-ab7747.html
https://www.abcam.com/rantes-antibody-ab9783.html
https://www.abcam.com/il-1-beta-antibody-ab9722.html
https://www.abcam.com/il-1ra-antibody-epr6483-ab124962.html
https://www.abcam.com/cd68-antibody-ab125212.html
https://www.abcam.com/cd68-antibody-ed1-ab31630.html
https://www.abcam.com/histone-h3-phospho-s10-antibody-ab5176.html
https://www.abcam.com/histone-h3-antibody-nuclear-marker-and-chip-grade-ab1791.html
https://www.abcam.com/trpm7-antibody-c-terminal-ab135817.html
https://www.abcam.com/trpm7--trpm6-antibody-epr4582-ab109438.html
https://www.abcam.com/trpm7-antibody-n7425-ab85016.html
https://www.abcam.com/osteopontin-antibody-ab8448.html
https://www.scbt.com/p/alp-antibody-b-10
https://www.cellsignal.com/products/primary-antibodies/ikkb-d30c6-rabbit-mab/8943
https://www.cellsignal.com/products/primary-antibodies/ikka-3g12-mouse-mab/11930

https://www.cellsignal.com/products/primary-antibodies/phospho-ikba-ser32-14d4-rabbit-mab/2859
https://www.cellsignal.com/products/primary-antibodies/sapk-jnk-antibody/9252
https://www.cellsignal.com/products/primary-antibodies/phospho-sapk-jnk-thr183-tyr185-81e11-rabbit-mab/4668
https://www.cellsignal.com/products/primary-antibodies/ikba-l35a5-mouse-mab-amino-terminal-antigen/4814
https://www.cellsignal.com/products/primary-antibodies/sf2-asf-antibody/8241
https://www.scbt.com/p/nfatc1-antibody-7a6
hhttps://www.cellsignal.com/products/primary-antibodies/b-actin-d6a8-rabbit-mab/8457

Eukaryotic cell lines

Policy information about cell lines

Cell line source(s)	THP-1 (ATCC)
Authentication	ATCC uses morphology, karyotyping, and PCR based approaches to confirm the identity of human cell lines and to rule out both intra- and interspecies contamination. These include an assay to detect species specific variants of the cytochrome C oxidase I gene (COI analysis) to rule out inter-species contamination and short tandem repeat (STR) profiling to distinguish between individual human cell lines and rule out intra-species contamination (https://www.atcc.org/CellAuthenticationMatters.aspx). The cell line used in this paper has been passaged separately from other cell lines in the lab. The cytokine profiles shown in Fig.4g indicated that this cell line was unlikely to be misidentified.
Mycoplasma contamination	The cell line not tested for mycoplasma contamination.
Commonly misidentified lines (See ICLAC register)	THP-1 is NOT on the list of commonly misidentified lines

Animals and other organisms

Policy information about studies involving animals; ARRIVE guidelines recommended for reporting animal research

Laboratory animals	Sprague Dawley Rat, female, 6-8-week old
Wild animals	Not applicable
Field-collected samples	Not applicable
Ethics oversight	All the animal procedures were performed in accordance with a protocol approved by the Committee on the Use of Live Animals in Teaching and Research (CULATR, HKU)

Note that full information on the approval of the study protocol must also be provided in the manuscript.

Flow Cytometry

Plots

Confirm that:

☒ The axis labels state the marker and fluorochrome used (e.g. CD4-FITC).

☒ The axis scales are clearly visible. Include numbers along axes only for bottom left plot of group (a 'group' is an analysis of identical markers).

☒ All plots are contour plots with outliers or pseudocolor plots.

☒ A numerical value for number of cells or percentage (with statistics) is provided.

Methodology

Sample preparation	Differentiated macrophages were detached with trypsin and washed with 1X PBS. For the detection of macrophage surface markers, cells were incubated with monoclonal mouse anti-human antibodies CD163-Cy5.5, CD206-FITC, and CD80-FITC (BD Biosciences, USA), or relevant isotypes (BD Biosciences) for 1 hr at 4 ℃ in dark. After washing with 1X PBS, the fluorescence was compared to isotypes with 10,0000 events recorded.
Instrument	FACSCantoII Analyzer (BD Biosciences, USA)
Software	Flowjo software, version 10 (Tree Star, USA)
Cell population abundance	Cell populations abundance is presented on the relevant figures.

Gating strategy	The total THP1 cells were first gated by forward and side scatter, CD163, CD206 and CD80 positive cells were further gated using the same gating strategies defined by THP1 cells fixed identically to the experimental groups and stained with immunoglobulin-matched FITC- or Cy5.5-conjugated isotype control

☒ Tick this box to confirm that a figure exemplifying the gating strategy is provided in the Supplementary Information.
